# Job Stress and Presenteeism among Chinese Healthcare Workers: The Mediating Effects of Affective Commitment

**DOI:** 10.3390/ijerph14090978

**Published:** 2017-08-29

**Authors:** Tianan Yang, Yina Guo, Mingxu Ma, Yaxin Li, Huilin Tian, Jianwei Deng

**Affiliations:** 1 School of Management and Economics, Beijing Institute of Technology, Beijing 100081, China; tianan.yang@bit.edu.cn (T.Y.); 13142234583@163.com (Y.G.); 13146043697@163.com (M.M.); liyaxinyayaya@163.com (Y.L.); 18810981060@163.com (H.T.); 2 Sustainable Development Research Institute for Economy and Society of Beijing, Beijing 100081, China

**Keywords:** healthcare workers, challenge stress, hindrance stress, affective commitment, presenteeism, public service quality

## Abstract

Background: Presenteeism affects the performance of healthcare workers. This study examined associations between job stress, affective commitment, and presenteeism among healthcare workers. Methods: To investigate the relationship between job stress, affective commitment, and presenteeism, structural equation modeling was used to analyze a sample of 1392 healthcare workers from 11 Class A tertiary hospitals in eastern, central, and western China. The mediating effect of affective commitment on the association between job stress and presenteeism was examined with the Sobel test. Results: Job stress was high and the level of presenteeism was moderate among healthcare workers. Challenge stress and hindrance stress were strongly correlated (β = 0.62; *p* < 0.05). Affective commitment was significantly and directly inversely correlated with presenteeism (β = −0.27; *p* < 0.001). Challenge stress was significantly positively correlated with affective commitment (β = 0.15; *p* < 0.001) but not with presenteeism. Hindrance stress was significantly inversely correlated with affective commitment (β = −0.40; *p* < 0.001) but was significantly positively correlated with presenteeism (β = 0.26; *p* < 0.001). Conclusions: This study provides important empirical data on presenteeism among healthcare workers. Presenteeism can be addressed by increasing affective commitment and challenge stress and by limiting hindrance stress among healthcare workers in China.

## 1. Introduction

Definitions of presenteeism vary [1]. In the beginning, most studies focused on this behavior, and defined it as “attending work while ill” [1] or “going to work despite feeling unhealthy” [2,3]. However, as interest in presenteeism has increased, scholars have found a negative impact of presenteeism on the productivity of organizations, and greater losses to the organizations than from absenteeism [4,5]. In this context, scholars began to define presenteeism from both the aspects of health and the productivity loss due to presenteeism, such as “reduced productivity due to health and other events that distract employees from full capacity” [1,4,6,7]. Moreover, studies also have found that, in addition to sickness presenteeism, presenteeism now also refers to chatting, procrastination, or surfing the internet, which decrease employee performance [1,4,6,8]. Employees who are present at work but work with low efficiency while receiving a full salary might reduce the productivity of enterprises or organizations [9,10,11]. Subsequently, empirical studies of the perceived ability to work scale (PAWS) validated it as a measure of this form of presenteeism [12,13,14,15], as did a large national survey in the United States [15]. Because some recent studies have defined presenteeism as potential productivity loss in the workplace due to health and other events [12,14,16,17], we used this definition in the present study.

Past studies showed that job stress is a critical factor in presenteeism [18,19,20,21]. However, additional empirical evidence would allow us to disentangle the effects of different types of job stress on productivity-related outcomes. In this study, we classify job stress as challenge stress and hindrance stress. Challenge stress refers to job stress that individuals feel they can overcome and that benefits their career development, such as job load, job responsibility, and time urgency. Hindrance stress refers to stress that individuals feel they cannot overcome and that prevents their career development, such as role conflict, organizational politics, and work insecurity [22]. Both challenge stress and hindrance stress can have adverse effects on emotional well-being [23,24], but the mechanism of such effects differs. In most cases, challenge stress will have a positive effect on a person, as it stimulates desirable emotions and encourages people to solve problems in a positive way [24]. Conversely, hindrance stress will have adverse effects. Lepine and Podsakoff [25,26] held that challenge stress has positive effects on performance and commitment, while hindrance stress has adverse effects. Although performance is a central concern in enterprises and organizations, few studies have examined the differential effects of challenge stress and hindrance stress on presenteeism and other productivity-related outcomes. Increased study of these different types of job stress in specific cultural and occupation contexts could improve understanding of the effects of job stress on presenteeism.

Job stress is a strong predictor of presenteeism; however, only a few studies have examined mechanisms that mediate between job stress and presenteeism in our definition. Indeed, only health was mainly considered as a mediator of presenteeism, defined in various definitions, in previous studies [12,14,27]. Nevertheless, affective commitment is also important in presenteeism [28,29,30] and job stress [31,32,33]. Affective commitment is a critical dimension of organizational commitment and reflects employee values, goal commitment, and affective dependence on the organization [31], which was considered an important antecedent variable of presenteeism [28] and an important outcome variable of stress [34]. Social exchange theory [35] provides further explanations. An interdependent social exchange relationship between employees and organizations is the result of an exchange of interests, which is rooted in communication and support among employees. Job stress involves communication and responses from the organization, supervisors, colleagues, and staff; e.g., the support of the organization, assistance of the supervisor, and other activities. These activities accumulate over time and strengthen the relationship between organization and employees. Then, the emotional dependence of employees on the organization, referred to as affective commitment, is formed. Affective commitment can encourage employees to increase behaviors that are beneficial to organizations, such as fulfilling their job responsibilities or expanding the scope of work, and can improve performance and decrease presenteeism. These behaviors can enhance an employee’s work ability and reduce presenteeism. Thus, we tested the hypothesis that affective commitment reduces presenteeism.

**Figure 1:**
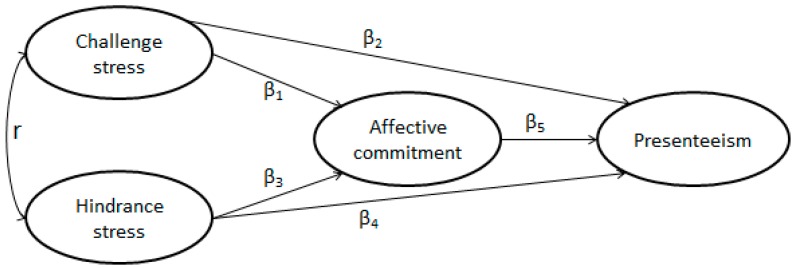
Proposed model of how challenge stress, hindrance stress, and affective commitment affect presenteeism.

Previous studies reported that the burden of treating the dying and injured leads to high presenteeism and job stress among providers of health services, such as healthcare workers [11,36,37,38]. Presenteeism and job stress among healthcare workers increase burnout and medical errors and endanger the safety of patients [39]; thus, the need to investigate this group is great. Increases in health problems and other forms of job stress in China have increased the prevalence of mental illness among Chinese healthcare workers, diminished their enthusiasm for work, and led to an indifferent attitude to patients and increased presenteeism. Such conditions degrade the quality of health services and hospital performance [40]. Therefore, in this study we enrolled a sample of Chinese healthcare workers and examined the mediating effects of affective commitment on the association between job stress and presenteeism among them. Figure 1 illustrates the proposed integration of these components.

## 2. Methods

### 2.1. Data Source

This cross-sectional study analyzed data from 1392 healthcare workers employed in Class A tertiary hospitals in eastern, central, and western China in 2016 with ethics approval (including doctors, nurses, pharmacists, medical technicians, and administrative personnel in hospitals and clinics; response rate 92%). Tertiary hospitals are comprehensive or general hospitals at the city, provincial, or national level and have more than 500 beds. They provide specialist health services, are important in medical education and scientific research, and serve as medical hubs providing care to multiple regions. Most medical services are provided by tertiary and secondary hospitals, and workers at Class A tertiary hospitals are more likely to report high job stress [41]. We thus selected Class A tertiary hospitals as the focus of our research. The survey assessed individual characteristics, job stress, affective commitment, and presenteeism. Because the ratio of Class A tertiary hospitals in eastern, central, and western China is 5.2:3.6:2.6, we used this ratio of the number of Class A tertiary hospitals in these three regions (5:3:3) to a randomly-selected five (Beijing, Guangzhou, Haikou, Shanghai, Xiamen), three (Wuhan, Changchun, Zhengzhou), and three (Chongqing, Kashi, Xi’an) hospitals, respectively, from these regions in this study. To ensure data integrity and objectivity, participants were selected by using random sampling according to geographical area, employee number, and job title. 

### 2.2. Variables and Instruments

Presenteeism was assessed with the four-item perceived ability to work scale (PAWS), a reliable and valid instrument for measuring perceived productivity loss that has acceptable psychometric properties [13,15,19]. The question, “How many points would you give your current ability to work?” (Table 1), asks respondents to rate their perceived ability on a scale from 0 to 10 (0 = cannot currently work at all; 10 = work ability is currently at its lifetime best). The scale was shown to have high reliability (α = 0.89) and acceptable psychometric properties. To improve intuitive understanding of the score, we changed its directionality by subtracting the original scores from 10. Thus, higher scores indicate greater presenteeism.

Job stress was measured by the challenge and hindrance-related self-reported stress (C-HSS) scale of 11 items. For example, the item, “The number of projects and/or assignments I have” (Table 1), evaluates challenge stress and hindrance stress [22] by using a five-point Likert scale (1 = no stress; 5 = great stress). Higher values reflect greater job stress. The C-HSS scale was shown to have high reliability (α = 0.87–0.75) in the present study.

Affective commitment was measured with a 15-item scale developed by Mowday, Steers, and Porter (1979). For example, the item, “I am willing to put in a great deal of effort beyond that normally expected in order to help this organization be successful” (Table 1) [42] asks respondents to rate their satisfaction with the organization on a scale from 1 (“strongly disagree”) to 7 (“strongly agree”). Higher values reflect greater affective commitment. The scale was shown to have high reliability (α = 0.85).

The Chinese versions of all the instruments were previously validated [43]. The individual characteristics analyzed included age, sex, education, job title, job experience, department, and seniority.

### 2.3. Statistical Analysis

SPSS20.0 and AMOS20.0 were used for the statistical analyses, which comprised descriptive analysis, correlation analysis, and path analysis. Structural equation modeling (SEM) analysis was used to examine relationships among challenge stress, hindrance stress, affective commitment, and presenteeism.

In SEM, four latent variables—presenteeism, challenge stress, hindrance stress, and affective commitment—were first constructed by using the PAWS indicators, namely, the C-HSS and measurement of organizational commitment scale. All these indicators have been examined whether model fit data well. Before imputing these indicators into the SEM, correlation analysis was used to determine the significance of correlations between presenteeism, challenge stress, hindrance stress, and affective commitment.

SEM can determine the effect relationship among variables, which can be classified as direct or indirect [44,45]. The effect is expressed by path coefficient beta (β). A direct effect is a relationship between two variables: the direct effect of challenge stress on affective commitment is β_1_ in Figure 1. An indirect effect is present when a variable affects another variable through an intermediate variable: the indirect effect of challenge stress on presenteeism is β_1_ × β_5_. Sobel test was used to examine the significance of mediated effects [46,47,48]. 

To determine if standardized regression coefficients (β) differed by subgroup, we conducted subgroup analyses of two age groups and two job-title groups. To ensure that the two subgroups were of equal size, age was categorized as old (31 years or older) and young (30 years or younger), based on the median (30 years) of the final sample. Job title was classified as early career group (trainee or primary) and mid/late career group (middle and senior).

## 3. Results

### 3.1. Demographic Characteristics of Participants

Table 2 shows the demographic characteristics of the healthcare workers. Demographic information was missing for a few participants (3.3–5.9%). Among the 1392 participants, 21.3% were men and 74.5% were women; 42.3% were nurses, 30.5% were clinicians, 11.4% were medical technicians, 8.5% were managers, and 1.8% were pharmacists. With respect to age group, 38.6% were age 25–30 years and only 0.2% were age 60 years or older. With respect to education level, 41.5% had earned an undergraduate degree, 21.5% had earned a master’s degree, 21.1% had graduated from junior college, and 7.9% had earned a doctorate. Most respondents (53.1%) had an early-career position, 27.6% had a mid-career position, and 8.6% were senior employees. Overall, 24.5% of participants had worked less than 3 years, 25.5% had worked 3–5 years, and 22.1% had worked 6–10 years. Pediatrics (18.7%), internal medicine (16.5%), and surgery (16.2%) were the most common departmental affiliations; only 0.9% of participants were in the oncology department (Table 2).

Table 1 shows the results (mean (M) and SD) for presenteeism, challenge stress, hindrance stress, and affective commitment items. The means for the four presenteeism items were relatively low and similar. They ranged from 7.25 (“Thinking about the mental demands of your job, how do you rate your current ability to meet those demands?”; SD = 1.79) to 7.63 (“How many points would you give your current ability to work?”; SD = 1.57). The mean for the challenge stress items was higher than that for hindrance stress. The third challenge stress item (“The volume of work that must be accomplished in the allotted time”) had the lowest score (M = 3.36, SD = 0.85), and the fifth item had the highest score (M = 3.56, SD = 0.87). The second hindrance stress item had the lowest score (M = 2.38, SD = 1.05), and the fourth item had the highest score (M = 3.04, SD = 1.05). The means for the fifteen affective commitment items were high. They ranged from 4.17 (“I would accept almost any type of job assignment in order to keep working for this organization”; SD = 1.61) to 5.41 (“I really care about the fate of this organization”; SD = 1.41).

### 3.2. Correlations between Presenteeism, Challenge Stress, Hindrance Stress, and Affective Commitment

Correlation coefficients (r) showed positive correlations between items within the same construct (Table 3). Presenteeism was significantly positively correlated with challenge stress and hindrance stress (r = 0.20–0.32). Presenteeism was significantly inversely correlated with affective commitment (r = −0.33). Affective commitment was significantly inversely correlated with challenge stress and hindrance stress (r = −0.09 to −0.33). There was also a significant positive correlation between challenge stress and hindrance stress (r = 0.53).

### 3.3. SEM

**Figure 2:**
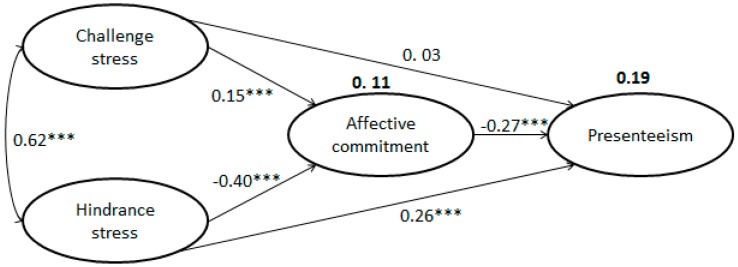
Final model illustrating how challenge stress and hindrance stress influence affective commitment and presenteeism (numbers not in bold are standardized regression coefficients and numbers in bold explain variability; chi square, 773.373; degrees of freedom, 0.99, *p* < 0.001; root mean square error of approximation, 0.068; goodness-of-fit index, 0.935; normed fit index, 0.951; comparative fit index, 0.945; *** *p* < 0.001).

Before SEM, the analysis of the measurement model showed that our model fits the data well, because the values for the goodness-of-fit index and comparative fit index of each measurement model were all between 0.916 and 0.989. In the final model, the affective commitment was directly inversely associated with presenteeism (β = −0.27; *p* < 0.001). Hindrance stress was moderately and significantly positively associated with presenteeism (β = 0.26; *p* < 0.001), but the path from challenge stress to presenteeism was not significant (β = 0.03; *p* > 0.05). There was a direct positive association between challenge stress and hindrance stress (β = 0.62; *p* < 0.001). Challenge stress was significantly positively associated with affective commitment (β = 0.15; *p* < 0.001), and hindrance stress was significantly inversely associated with affective commitment (β = −0.40; *p* < 0.001). Challenge stress and hindrance stress explained 11% of the variability in affective commitment. Challenge stress, hindrance stress, and affective commitment explained 19% of variability in presenteeism. Criteria for fitness, such as the root mean square error of approximation, goodness-of-fit index, comparative fit index, and normed fit index, indicated that the revised model was more appropriate (Figure 2). 

We noted significant indirect effects between challenge stress and presenteeism (Sobel z = −3.66; *p* < 0.001) and between hindrance stress and presenteeism (Sobel z = 7.11; *p* < 0.001), which were significantly mediated by affective commitment.

Subgroup analyses showed that the effect of challenge stress on presenteeism was different in the final model. Challenge stress did not significantly affect presenteeism (Table 4). Interestingly, the impact of challenge stress on affective commitment was only significant at *p* < 0.05 in older and mid/late career workers. The difference between the confirmatory factors analysis of our models of varying levels of measurement invariance is less than 0.01, which suggested that invariance might be tenable across old/young and early/mid-late groups [49,50].

## 4. Discussion

As expected in the initial model, hindrance stress impaired the affective commitment of Chinese healthcare workers, while challenge stress increased it. Hindrance stress significantly increased presenteeism among healthcare workers, but the increase attributable to challenge stress was not significant. Affective commitment was associated with a significant adverse impact on presenteeism. These associations were partially mediated by affective commitment.

We investigated the effects of different types of job stress on affective commitment and presenteeism and found that hindrance stress adversely affects affective commitment and presenteeism and that challenge stress has a significant direct negative effect on affective commitment but not presenteeism. These results partially confirm those of previous studies, most of which focused on the influence of job stress on affective commitment but only a few [26] attempt to examine the differential effects of job stress, challenge stress, and hindrance stress. Prior studies concluded that job stress is important in demission, i.e., greater job stress was associated with lower affective commitment, higher turnover, and presenteeism [32,51,52]. However, although job stress clearly affects behavior and other relative outcomes, the effects might differ for challenge and hindrance stress. Hindrance stress is related to excessive demands [53] which might reduce job satisfaction and affective commitment [26], a phenomenon explained by the theory of cognitive interaction [54]. When faced with challenge stress, employees choose a problem-oriented strategy, such as working hard to overcome stress and accomplish higher job requirements. This might encourage worker loyalty to the organization [27], satisfaction [23], and affective commitment. Studies found that challenge stress has a positive effect on employees' organizational commitment and loyalty [23,26]. Conversely, negative emotions due to hindrance stress may lead to go-slow and counterproductive behaviors, and presenteeism will gradually increase. The result is consistent with existing evidence [55,56,57], which shows that job stress has a significant positive effect on presenteeism. Our findings indicate that employees may be more loyal under conditions of challenge stress and might report a potential productivity loss when faced with hindrance stress. 

Moreover, because of the complexity of the healthcare environment, challenge stress did not directly significantly affect presenteeism, which was confirmed in a multigroup analysis. This finding is consistent with the results of previous studies, which called for further investigation of why the impact of challenge stress was weaker than that of hindrance [25,26]. To our knowledge, most healthcare workers in China are extremely overworked [58]. The work day is usually 10 h or longer. In addition, incomes are lower for Chinese healthcare workers than for their counterparts in Europe and the United States, which do not have the same high workloads. Healthcare workers in China also have health problems at work, which further affect their emotional well-being [59,60,61]. Moreover, the amount and extent of job stress among Chinese healthcare workers are extremely high, and healthcare workers are exceptionally overworked. In this context, challenge stress is unlikely to have positive effects on healthcare workers. Instead, increases in job stress—regardless of whether it is challenge or hindrance stress—will eventually lead to negative consequences, and ultimately to burnout, turnover, or lower performance. Furthermore, our results of multigroup analysis, although association between challenge stressors and presenteeism for the two groups “old” (*p* = 0.08) and “mid/late career” (*p* = 0.06) employees was suggestive, did not achieve significant level. This indicates that challenge stress might have a positive impact on presenteeism among these participants. Challenge stress might not generate its positive outcomes when the job stress of Chinese healthcare workers is extremely beyond their tolerance. We suspect that this explains why challenge stress did not have a significant effect on presenteeism in our study. 

In conclusion, our study provides empirical evidence that the impact of challenge stress is weaker than that of hindrance stress, because of the complexity of the healthcare environment and the long history of overwork. Thus, policymakers should be made aware of the intense stress on healthcare workers and focus on interventions targeting hindrance stress—such as reasonable shift work, reforming pay structures, balancing the work–family interface [62] and adequate treatment of healthcare problems. For instance, studies have described the negative effects of violence against Chinese healthcare workers and unfair promotion and career advancement in Chinese public hospitals [63,64,65]. A patient feedback system in hospitals could be introduced to address patient concerns and chronic discrimination. Such a system might prevent conflicts and improve career development. Later efforts could address unfairness in promotion and reduction of excess workloads, thereby improving job conditions and employees’ affective commitment. Most importantly, the analysis of the interrelationships between challenge stress, affective commitment, and presenteeism suggests that Chinese healthcare workers have long been continually exposed to job stress, both challenge and hindrance stress. Under such circumstances, challenge stress can impair the performance and health of healthcare workers. To effectively intervene their job stress and promote their health and performances, proposed challenging work in Chinese hospitals might be completed when healthcare workers have tried their best or else they will increase anxiety among workers or decrease their confidence. Future studies should investigate methods to control challenge stress and identify the critical point between challenge stress and hindrance stress.

Our present findings show that affective commitment had a significant negative effect on presenteeism. Using our concept of presenteeism, we observed that affective commitment has a negative effect on presenteeism, which was confirmed by empirical research. Our finding is consistent with some of the previous studies. In a study of government workers, Taifor et al. found that affective commitment had a negative effect on presenteeism [66]. High affective commitment can enhance employee loyalty and sense of belonging to an enterprise. Employees will consciously restrain their behavior and improve their work performance [67]. We found that affective commitment negatively affects presenteeism, which may be related to Chinese culture and institutions. In Chinese traditional culture and values, loyalty, progress, and dedication are highly desirable [68], so healthcare workers in China are proud of their dedication and responsibility [69,70]. They typically have a strong sense of loyalty and commitment to organizations that provide sufficient resources. Moreover, Chinese hospitals have a unique system to control personnel establishment, called *bianzhi*. Healthcare workers in the *bianzhi* system can sign a contract without a fixed term with a hospital, benefit from national welfare services, and do not need to worry about losing their job unless they commit a major error. Hence, healthcare workers might have greater loyalty to hospitals. They restrain their behavior at work, work hard to remain at the organization, and maintain their productivity. Our findings indicate that hospital managers might increase the level of affective commitment, reduce presenteeism, and boost performance among healthcare workers by trying to improve job conditions and the psychosocial work environment. Managers should also attempt to improve the psychological work environment and opportunities for highly capable employees not in the *bianzhi* system.

Another important finding of this study is that affective commitment mediates the effects of hindrance stress and presenteeism. Previous studies considered only health as an important mediator between job stress and presenteeism [12,27]. Our results yield insight into how job stress affects presenteeism through affective commitment. A future study could select affective commitment as a factor in the design of presenteeism in hospitals. The present results suggest that affective commitment and employee retention are likely to be higher in public hospitals when employees receive sufficient organizational resources to deal with all types of stress and promote their own professional growth. Hospitals could implement training programs on topics such as coping skills, time management, and career planning.

Finally, the present findings help refine the concept of presenteeism from the perspective of productivity loss and highlight avenues for future empirical research on presenteeism. Presenteeism was originally defined as “attending work while ill” [1]. This definition concerns the relationship between presenteeism and health problems and considered health-related factors as the only indicators of presenteeism [11]. In the present study, we defined presenteeism from the perspective of productivity loss. This concept includes not only behaviors such as reporting for work when ill, but also behaviors such as inadequate dedication to work because of personal problems or office politics. This definition of presenteeism includes behaviors that hurt an organization because employees cannot work at high efficiency but receive full pay without full productivity. 

## 5. Limitations

This study has limitations that warrant mention. First, we only recruited healthcare workers from Chinese Class A tertiary hospitals and excluded those from primary and secondary hospitals, which limits the generalizability and robustness of our conclusions. Nevertheless, our findings should be helpful for policymakers in countries or regions with large inequalities in resource allocation for health services. Second, job stress, affective commitment and presenteeism were all self-reported; thus, measurement of productivity loss may be subjective and lead to further bias. Future studies should take both subjective and objective data into account in their study design, obtain measures of predictor and criterion variables from different sources, or balance positive and negative items [71]. Third, challenge stress and hindrance stress are not clearly differentiated. Cognitive interaction theory maintains that job stress comes from the interaction between an individual and his/her environment. However, some external variables that affect stress cognition influence the formation and consequences of stress but were not considered in our analysis. Further research should improve measurement and conceptualization of job stress and its classification. Fourth, our study was conducted in the first year of a cohort study. The relationships between challenge stress, hindrance stress, affective commitment, and presenteeism should be examined in a future longitudinal study. Finally, we did not consider both the positive and negative aspects of presenteeism and job stress in this study. This also limits the generalizability of our model and conclusions. 

## 6. Conclusions

Although healthcare workers are the key to improving healthcare quality, Chinese healthcare workers are exposed to high job stress, severe health problems, and long-term overwork. This has led to poor affective commitment and increased presenteeism, which has degraded the quality of health services and performance at hospitals. To cope with these challenges in Chinese hospitals, it is vital to limit presenteeism and ensure delivery of medical quality by fostering and maintaining a higher level of affective commitment among healthcare workers. Future policies might focus on reducing presenteeism by decreasing job stress among healthcare workers, by improving work conditions, and by offering more opportunities to enhance affective commitment of workers.

## Figures and Tables

**Table 1:** Means (SD) for presenteeism (P), challenge stress (CS), hindrance stress (HS), and affective commitment (AC) items.

Variables	Items	Mead	SD
Presenteeism (0–10)	P1. How many points would you give your current ability to work?	2.37	1.57
	P2. Thinking about the physical demands of your job, how do you rate your current ability to meet those demands?	2.56	1.65
	P3. Thinking about the mental demands of your job, how do you rate your current ability to meet those demands?	2.75	1.79
	P4. Thinking about the interpersonal demands of your job, how do you rate your current ability to meet those demands?	2.66	1.73
Challenge stress (1–5)	CS1. The number of projects and or assignments I have.	3.48	0.87
	CS2. The amount of time I spend at work.	3.50	0.85
	CS3. The volume of work that must be accomplished in the allotted time.	3.36	0.88
	CS4. Time pressures I experience.	3.45	0.88
	CS5. The amount of responsibility I have.	3.56	0.87
	CS6. The scope of responsibility my position entails.	3.49	0.89
Hindrance stress (1–5)	HS1. The degree to which politics rather than performance affects organizational decisions.	2.85	1.04
	HS2. The inability to clearly understand what is expected of me on the job.	2.38	1.05
	HS3. The amount of red tape I need to go through to get my job done.	3.01	1.00
	HS4. The lack of job security I have.	2.98	1.08
	HS5. The degree to which my career seems stalled.	3.04	1.05
Affective commitment (1–7)	AC1. I am willing to put in a great deal of effort beyond that normally expected in order to help this organization be successful.	4.63	1.49
	AC2. I talk up this organization to my friends as a great organization to work for.	4.81	1.49
	AC3. I feel very little loyalty to this organization. (R)	5.14	1.60
	AC4. I would accept almost any type of job assignment in order to keep working for this organization.	4.17	1.61
	AC5. I find that my values and the organization’s values are very similar.	4.41	1.56
	AC6. I am proud to tell others that I am part of this organization.	4.91	1.69
	AC7. I could just as well be working for a different organization as long as the type of work was similar. (R)	4.22	1.54
	AC8. This organization really inspires the very best in me in the way of job performance.	4.34	1.65
	AC9. It would take very little change in my present circumstances to cause me to leave this organization. (R)	5.19	1.46
	AC10. I am extremely glad that I chose this organization to work for over others I was considering at the time I joined.	5.05	1.49
	AC11. There’s not too much to be gained by sticking with this organization indefinitely. (R)	4.40	1.70
	AC12. Often, I find it difficult to agree with this organization’s policies on important matters relating to its employees. (R)	4.11	1.68
	AC13. I really care about the fate of this organization.	5.41	1.41
	AC14. For me, this is the best of all possible organizations for which to work.	4.90	1.50
	AC15. Deciding to work for this organization was a definite mistake on my part. (R)	4.97	1.85

An “R” denotes a negatively phrased and reverse scored item.

**Table 2:** Demographic characteristics of the sample of healthcare workers.

Characteristics	Sample (*n* = 1392)	Percent (%)
Sex		
Male	297	21.3%
Female	1037	74.5%
Age (years)		
~25	189	13.6%
25~30	538	38.6%
31~35	302	21.7%
36~40	128	9.2%
41~50	138	9.9%
51~55	40	2.9%
56~60	8	0.6%
60~	3	0.2%
Post		
Clinician	425	30.5%
Nurse	589	42.3%
Management	119	8.5%
medical technicians	158	11.4%
Pharmacist	25	1.8%
Education		
Under degree	53	3.8%
Junior college	295	21.2%
Undergraduate	577	41.5%
Master	299	21.5%
Doctor	110	7.9%
Title		
Trainee	67	4.8%
Primary	739	53.1%
Middle	384	27.6%
Senior	120	8.6%
Seniority (years)		
~3	341	24.5%
3~5	355	25.5%
6~10	307	22.1%
11~20	193	13.9%
20~	140	10.1%
Department		
Physician	229	16.5%
Surgeon	226	16.2%
Obstetrics/gynecology	132	9.5%
Pediatrics	260	18.7%
Chinese Medicine	102	7.3%
Oncology	12	0.9%
Other clinical departments	84	6.0%
Medical technology	181	13.0%
Administration and Logistics	90	6.5%

**Table 3:** Intercorrelations between presenteeism (P), challenge stress (CS), hindrance stress (HS), and affective commitment (AC) items (** *p* < 0.01).

Variables (Mean (M), SD)	Items			
	P	CS	HS	AC
P (2.59, 1.5)	1			
CS (3.47, 0.75)	0.20 **	1		
HS (2.85, 0.81)	0.32 **	0.53 **	1	
AC (4.70, 0.93)	−0. 33 **	−0.09 **	−0.28 **	1

**Table 4:** Standardized regression coefficients (β) with p values for the components of subgroup analyses.

Path	Young (under 30 Years, *n* = 727)		Old (over 31 Years, *n* = 619)		Early Career (*n* = 806)		Mid/Late Career (*n* = 504)	
	β	*p* Value	β	*p* Value	β	*p* Value	β	*p* Value
CS to AC	0.13	**	0.17	*	0.15	**	0.13	*
HS to AC	−0.40	***	−0.41	***	−0.44	***	−0.38	***
AC to P	−0.22	***	−0.31	***	−0.24	***	−0.34	***
CS to P	−0.04	0.44	0.10	0.08	−0.03	0.47	0.12	0.06
HS to P	0.31	***	0.21	***	0.29	***	0.20	**
CS to HS	0.59	***	0.66	***	0.60	***	0.65	***

CS, challenge stress; HS, hindrance stress; AC, affective commitment; P, presenteeism; * significant at *p* < 0.05; ** significant at *p* < 0.01; *** significant at *p* < 0.001.
